# Medical and Philosophical News

**Published:** 1781-02

**Authors:** 


### SECTION III.

### Medical and Philosophical News.

THE Society of Natural Hiſtorians at Berlin have propoſed the following queſtion for a prize of twenty ducats: 1. "How "long is the virus producing hydrophobia ca-"pable of remaining in an animal, and what is "the length of time required for communicat-"ing it? 2. How long can this ſame virus "remain in the body without manifeſting itſelf? "From the moment the diſeaſe is communi-"cated, what are the moſt efficacious means of "obviating its effects?" The diſſertations on this ſubject are to be written in French or Latin, and ſent to M. Otho, ſecretary to the Society at Berlin, on or before the 27th of December 1781.

